# Brain Rot as an Emerging Concept in Nursing: A Concept Analysis for Nursing Management

**DOI:** 10.1155/jonm/7646151

**Published:** 2026-10-21

**Authors:** Ebtsam Aly Abou Hashish, Reham Yasin

**Affiliations:** ^1^ College of Nursing Jeddah King Saud Bin Abdul-Aziz University for Health Sciences Jeddah Saudi Arabia ksau-hs.edu.sa; ^2^ King Abdullah International Medical Research Center Jeddah Saudi Arabia kaimrc.med.sa; ^3^ Ministry of National Guard Health Affairs Jeddah Saudi Arabia mngha.med.sa; ^4^ Faculty of Nursing Alexandria University Alexandria Egypt alexu.edu.eg

**Keywords:** brain rot, concept analysis, digital media, digital well-being, nursing management

## Abstract

**Aim(s):**

To clarify brain rot as an emerging concept, define its provisional boundaries, and examine its relevance to nursing management.

**Background:**

Brain rot describes perceived cognitive and psychological deterioration associated with recurrent consumption of trivial or undemanding digital content. Its status as a distinct construct, particularly in nursing, remains unestablished.

**Evaluation:**

Walker and Avant’s eight‐step concept analysis method guided a purposive, iterative analysis of 56 sources identified across seven databases and supplementary reference checking. Evidence from brain‐rot research, problematic digital use, cognition, technostress, and nursing work was compared to clarify attributes, boundaries, antecedents, consequences, and empirical referents.

**Key Issue(s):**

Five provisional attributes were identified: recurrent passive consumption of low‐quality digital content, cognitive disruption, emotional dysregulation, reduced control over engagement, and functional impairment. Temporal progression was treated as a testable course characteristic rather than an attribute. Occupational digital load, workload, shift fatigue, and limited recovery were classified as contextual vulnerabilities rather than causes. Nursing‐specific manifestations remain hypothetical.

**Conclusion(s):**

Brain rot is regarded as a provisional conceptual configuration requiring discriminant, incremental, and longitudinal validation against established constructs.

**Implications for Nursing Management:**

Nurse managers should address established technology‐related strain and recovery needs while supporting research; brain rot–specific screening, policy, or intervention is premature.

## 1. Introduction

Digital technologies have transformed nursing practice through electronic health records (EHRs), telehealth, mobile health applications, and artificial intelligence–supported systems [1–5]. These technologies can improve access, documentation, communication, and care coordination, yet poorly designed or burdensome systems may also contribute to interruption, cognitive load, digital fatigue, and technostress [6–10]. Nurses therefore work in digitally intensive environments while sustaining therapeutic communication, clinical reasoning, evidence‐based decision‐making, and safe care [11, 12]. EHR documentation burden and workflow disruption are established concerns [7, 8, 13], but these occupational technology demands should not be interpreted as evidence that brain rot occurs in nurses.

Outside work, nurses and nursing students also use social media and other digital platforms for communication, information, and leisure [14]. Across broader populations, problematic social media and internet use has been associated with psychological distress, reduced control over use, and functional interference [15, 16]. In December 2024, Oxford University Press selected “brain rot” as its Word of the Year, reflecting growing concern about perceived cognitive and psychological deterioration associated with overconsumption of trivial or intellectually undemanding online material [17]. Emerging studies have subsequently examined the term in student and younger populations [18–22]. Broader research on heavy digital media use and media multitasking reports associations with attention, executive control, and cognitive functioning [23–25]. These findings provide a rationale for conceptual inquiry, but they neither establish a specific neurobiological mechanism for brain rot nor demonstrate the construct in nurses.

### 1.1. Concept Selection and Purpose of Analysis

Guided by Walker and Avant’s eight‐step framework [26], this analysis treats brain rot as a provisional, testable conceptual configuration rather than a diagnosis. The purpose was to clarify current uses of the term; identify proposed defining attributes; distinguish it from problematic social media use, internet addiction, technostress, and burnout; separate antecedents and contextual vulnerabilities from potential consequences; identify empirical referents; and outline proportionate priorities for nursing research, education, leadership, and practice.

Nursing work includes mandatory digital documentation, shift work, occupational fatigue, emotional demands, and limited recovery opportunities [6–10, 27–30]. These established work conditions may plausibly influence cognitive recovery or the appeal of rapid recreational media after demanding shifts, but current evidence does not show that they cause brain rot. Their role in this analysis is therefore contextual: they are potential modifiers to examine when testing whether voluntary low‐quality digital consumption has explanatory value beyond established occupational stressors.

Accordingly, Walker and Avant’s [26] framework (Figure 1) was used to develop a provisional, testable conceptual configuration rather than a clinical diagnosis or established occupational syndrome.

**FIGURE 1:**
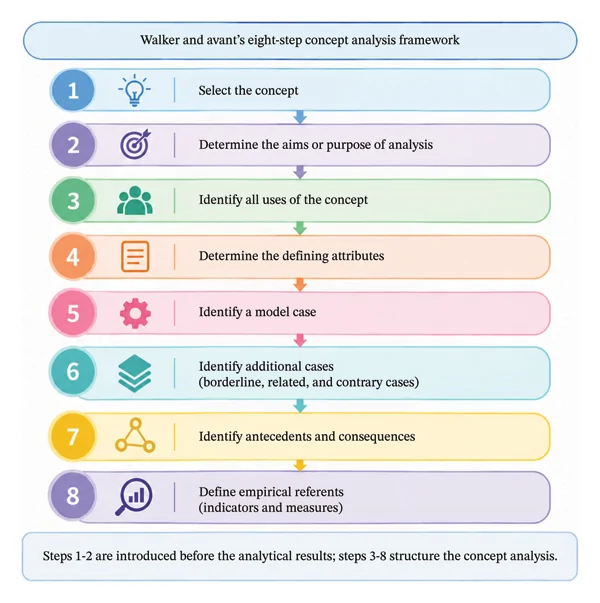
Walker and Avant’s eight‐step concept analysis framework. Original conceptual illustration developed by the authors from Walker and Avant [26].

## 2. Methods

### 2.1. Design

This study used Walker and Avant’s eight‐step concept analysis method [26]. Steps 1–2 are addressed in the Introduction and Steps 3–8 in the Results. Because the purpose was conceptual clarification rather than effect estimation, the review was purposive and concept‐driven rather than a systematic review.

### 2.2. Literature Review and Search Strategy

A purposive and iterative literature search was conducted from January through March 2026 across PubMed/MEDLINE, CINAHL, PsycINFO, Scopus, Web of Science, ERIC, and Google Scholar. The primary publication window was 2016–2025; directly relevant early‐2026 brain‐rot studies identified during the search were retained as emerging evidence, while earlier seminal sources were included when necessary for definition, theory, measurement, or historical context. Database searching was supplemented by reference‐list checking of highly relevant papers. Because the term brain rot is recent and nursing‐specific evidence is limited, the strategy deliberately combined the exact term with literature on adjacent cognitive, behavioral, digital‐health, and nursing‐work phenomena. The purpose was conceptual boundary development rather than estimation of prevalence or causal effects.

Search concepts were adapted to database syntax and combined using AND and OR. Core combinations included “brain rot”; “digital media” AND cognition; “social media” AND attention; “short‐form video” AND cognition; “excessive screen time” AND cognitive function; “internet addiction” OR “problematic social media use”; “digital fatigue” AND nursing; “smartphone addiction” AND healthcare workers; technostress AND nursing; “EHR burden” AND cognitive load; and “occupational cognitive health.” Searches were limited to English‐language sources where database filters were available. The same conceptual domains were used across databases, although exact syntax varied by platform.

### 2.3. Inclusion and Exclusion Criteria

Sources were eligible when they directly informed the definition, attributes, conceptual boundaries, antecedents, consequences, or measurement of brain rot or closely related digital‐use constructs. Peer‐reviewed empirical studies and reviews were prioritized; theoretical or conceptual papers, academic books, and authoritative institutional sources were retained when they provided seminal, definitional, historical, or professional context. Sources were excluded when they lacked conceptual relevance, addressed only technical system features without cognitive or behavioral implications, consisted of nonscholarly commentary without institutional authority, or were not retrievable in full text. Studies focused exclusively on children or adolescents were not used to make nursing‐specific claims; where a younger‐population source directly informed emerging brain‐rot terminology or measurement, its population limitation was made explicit.

### 2.4. Data Extraction and Analytical Process

For each included source, the authors charted the construct examined, population and setting, source type, definition or description, candidate attributes, antecedent conditions, reported or proposed outcomes, and empirical indicators. Evidence was mapped to Walker and Avant’s framework and compared iteratively across sources. Candidate attributes were retained when they recurred across conceptually relevant literature and contributed to boundary clarification. Nursing‐specific applications were mapped only when supported by nursing or healthcare literature; where direct nursing evidence was absent, the application was explicitly treated as theoretical extrapolation rather than an empirical finding.

Analysis continued until additional sources no longer changed the working attributes, conceptual boundaries, antecedent categories, or empirical referents. Formal risk‐of‐bias scoring was not undertaken because the objective was concept development rather than estimation of intervention effects. Instead, sources were critically considered according to conceptual relevance, population applicability, methodological characteristics, source type, publication status, and authority. Peer‐reviewed empirical and review literature was given greatest weight for substantive claims, whereas books and institutional sources were used principally for seminal, definitional, historical, or professional‐context purposes.

### 2.5. Case Construction

Model, borderline, related, and contrary cases were constructed to clarify conceptual boundaries [26]. They are hypothetical illustrations, not validated clinical presentations.

### 2.6. Final Sample Characteristics

The final analytic corpus comprised 56 sources: 27 empirical studies; 13 narrative or scoping reviews; 9 theoretical, conceptual, or concept‐analysis papers; 4 academic books; and 3 institutional or professional reports. Twenty‐one sources involved nurses, other healthcare professionals, or directly nursing‐relevant settings. The remaining sources involved adult, student, or general populations and were used to establish broader conceptual and behavioral foundations rather than to make nursing‐specific empirical claims. The selection process was purposive and concept‐driven; therefore, the 56 sources represent the analytic corpus rather than a systematic‐review sample from which prevalence or effect estimates can be inferred. Figure 2 summarizes this process and the source composition without presenting unsupported PRISMA‐style screening counts.

**FIGURE 2:**
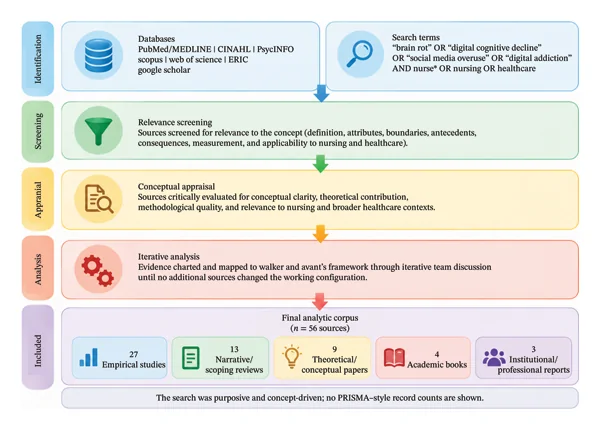
Concept‐driven literature identification, appraisal, and selection process. Note. No PRISMA‐style record counts are presented because the review was purposive and no systematic screening log was maintained.

## 3. Results

The analysis produced five provisional defining attributes together with antecedent conditions and contextual vulnerabilities, potential downstream consequences, illustrative cases, and empirical referents. Because direct empirical evidence in nurses remains limited, the results distinguish evidence derived from interdisciplinary literature from nursing‐specific applications that remain theoretical or require direct testing. Steps 1 and 2 were addressed in the Introduction; the Results therefore begin with Step 3.

### 3.1. Step 3: Identifying All Uses of the Concept

Oxford Languages defines brain rot as “the supposed deterioration of a person’s mental or intellectual state,” particularly from overconsumption of trivial or unchallenging online material [17]. Historically, Thoreau used the term in Walden to criticize passive consumption of intellectually undemanding material and diminished reflective thought [31]. Contemporary discourse uses the term for perceived mental fog, reduced attention, and cognitive fatigue after excessive engagement with low‐effort digital content [17]. Emerging scholarly studies have begun to examine brain rot directly, particularly among students and young adults [19–22]. Psychological and behavioral literature also describes adjacent constructs such as problematic social media use and internet addiction, characterized by reduced control and functional impairment [16, 32]. Taken together, these uses suggest a contemporary configuration centered on the quality and passivity of digital consumption, but they do not yet establish brain rot as a psychometrically distinct disorder or occupational condition.

### 3.2. Step 4: Determining the Defining Attributes

Five proposed defining attributes were retained after revision: recurrent passive consumption of low‐quality digital content, cognitive disruption, emotional dysregulation, reduced control over engagement, and functional impairment. These attributes are derived predominantly from non‐nursing and emerging brain‐rot literature and should not be interpreted as diagnostic criteria. Temporal progression was removed as a defining attribute because longitudinal evidence is insufficient; possible escalation over time is instead treated as a course characteristic to be tested prospectively. Nursing‐specific examples in Table 1 are illustrative applications intended to generate hypotheses, not established manifestations in nurses.

**TABLE 1:** Defining attributes of brain rot and illustrative nursing applications.

Attribute	Description	Illustrative nursing applications requiring testing
1. Passive low‐quality digital content consumption	Recurrent, low‐effort engagement with trivial, repetitive, or algorithmically curated content that requires little sustained cognitive effort. The attribute concerns the quality and passivity of consumption rather than screen time alone	Recreational short‐form scrolling during breaks or after shifts may be examined as an example; this pattern is not established as prevalent in nurses
2. Cognitive disruption	Attentional fragmentation, subjective mental fatigue, or difficulty sustaining cognitive effort within the broader proposed pattern. Specificity to brain rot has not been established	Potential difficulty sustaining attention, learning, or prolonged cognitive effort; requires direct measurement and control for sleep, workload, burnout, and technostress
3. Emotional dysregulation	Irritability, mood instability, or reduced emotional presence occurring with the broader pattern. These features are nonspecific and overlap with other occupational and mental‐health conditions	Possible reduced relational presence or irritability; should not be attributed to brain rot without excluding competing explanations such as stress, sleep loss, compassion fatigue, and burnout
4. Reduced control over engagement	Difficulty stopping or reducing recreational digital consumption despite intentions to disengage or recognized interference. This attribute overlaps with problematic‐use and addiction frameworks	Difficulty disengaging from recreational content or repeated use that displaces recovery; requires discriminant validation against problematic social media/internet use.
5. Functional impairment	Meaningful interference with sleep/recovery, learning, relationships, self‐care, or work functioning. Functional interference distinguishes the proposed configuration from high but controlled use	Potential effects on recovery, professional learning, relationships, or work functioning; medication‐error and patient‐safety effects are not established

Work‐related technology exposure requires separate treatment. EHR documentation, alerts, telehealth, electronic medication systems, and digital communication can contribute to technostress, interruption, and cognitive load, but they do not by themselves constitute the proposed brain‐rot pattern [7–10]. In this analysis, occupational digital load, shift‐related fatigue, workload, and limited recovery are contextual vulnerabilities that may shape recovery or recreational media habits. The focal behavioral exposure remains recurrent voluntary engagement with low‐quality digital content. This distinction is important because it prevents mandatory professional technology use from being misclassified as brain rot.

#### 3.2.1. Attribute 1: Excessive Consumption of Low‐Quality Digital Content

The first proposed attribute is recurrent, excessive, or difficult‐to‐regulate consumption of low‐quality digital content requiring little sustained cognitive effort. Examples in the broader literature and contemporary descriptions include algorithmically curated short‐form feeds, repetitive entertainment, and passive scrolling [17–22]. The emphasis is not screen time alone: prolonged digital engagement can be educational, relational, or professionally necessary. The proposed attribute instead concerns a pattern of low‐effort, passive content consumption that repeatedly captures attention. Recreational scrolling during breaks or after shifts is therefore only an illustrative nursing application; available nursing literature confirms social‐media engagement but does not establish that this pattern is prevalent or constitutes brain rot in nurses [14].

#### 3.2.2. Attribute 2: Cognitive Disruption

Cognitive disruption is proposed as a defining feature of the configuration and includes attentional fragmentation, subjective mental fatigue, reduced capacity for sustained cognitive effort, and possible working‐memory difficulty. Media‐multitasking and broader internet‐use research support investigation of these domains [23–25], but their specificity to brain rot and their occurrence in nurses remain unestablished. Neuroscientific findings concerning heavy media use may provide plausibility for studying cognitive mechanisms, yet they do not demonstrate that trivial digital content causes neurological deterioration or that a brain‐rot‐specific neurobiological pathway exists. In nursing research, cognitive disruption should therefore be measured directly and interpreted alongside sleep, workload, burnout, and technostress.

#### 3.2.3. Attribute 3: Emotional Dysregulation

Emotional dysregulation is proposed to include irritability, mood instability, reduced emotional presence, or difficulty regulating affect within the broader pattern. Studies of problematic digital and social media use report associations with anxiety, depressive symptoms, and psychological distress [15, 16], but these findings do not establish that brain rot causes emotional dysregulation. In nursing, emotional regulation is particularly relevant to therapeutic communication and relational care [11]; however, irritability, reduced empathy, and emotional exhaustion are nonspecific and may also reflect occupational stress, compassion fatigue, sleep loss, or burnout [27, 28, 33, 34]. Emotional indicators must therefore be interpreted as part of the proposed configuration rather than as brain‐rot–specific symptoms in isolation.

#### 3.2.4. Attribute 4: Reduced Control Over Engagement

Reduced control over engagement refers to difficulty stopping or reducing recreational digital consumption, automatic checking, urges to return to content, or continued use despite recognized interference. This attribute overlaps substantially with behavioral‐addiction and problematic‐use frameworks [16, 32]. It is retained because loss of voluntary control may help distinguish the proposed configuration from frequent but intentional digital use, but this overlap also creates an important validity challenge. Platform features such as infinite scroll, notifications, social feedback, and personalized recommendations are designed to sustain engagement [35, 36]; their contribution to brain rot remains theoretical and should not be interpreted as proof of addiction or a specific reward‐system mechanism.

#### 3.2.5. Course Characteristic Requiring Testing: Temporal Progression

Escalation over time is plausible from problematic‐use literature [16, 32], but current brain‐rot evidence is insufficient to establish temporal progression as a defining attribute. Longitudinal studies should test whether passive low‐quality content use becomes more frequent or harder to regulate and whether such change precedes functional impairment.

#### 3.2.6. Attribute 5: Functional Impairment

Functional impairment refers to meaningful interference with sleep or recovery, learning, relationships, self‐care, or work functioning. Its inclusion helps distinguish the proposed configuration from high but controlled digital use without meaningful interference [16, 32]. Problematic internet use has been associated with sleep problems [37], but functional impairment is nonspecific and may have multiple competing explanations. In nursing, the clinically important question is whether the proposed configuration predicts work or relational functioning after established factors such as workload, sleep loss, burnout, and technostress are accounted for. Current evidence does not establish that brain rot causes medication errors, missed deterioration, reduced care quality, or other patient‐safety outcomes.

The closest boundary challenge is problematic social media use and internet addiction because both include reduced control and functional impairment [16, 32]. Brain rot is hypothesized to add emphasis on passive or trivial content together with cognitive–emotional disruption; whether this provides discriminant or incremental validity remains unknown (Table 2). Burnout is a work‐related syndrome associated with chronic occupational stress [27], whereas technostress arises from technology‐related demands such as overload, complexity, uncertainty, or invasion [10, 38]. Either may occur without recurrent recreational consumption of low‐quality content, and the proposed brain‐rot configuration could theoretically occur outside the workplace. Compassion fatigue and attention‐deficit/hyperactivity disorder (ADHD) may share nonspecific features such as emotional exhaustion or attentional difficulty, but their established contexts differ [34, 39]. These comparisons clarify conceptual boundaries but do not establish brain rot as a distinct clinical entity.

**TABLE 2:** Differentiation of brain rot from related concepts.

Construct	Definition/focus	Typical trigger	Overlap	Key distinction from proposed brain rot	Status of distinction
Problematic social media use	Maladaptive or compulsive use of social‐platform activities associated with distress or functional impairment	Social media engagement	Reduced control, salience, impairment, and repeated checking	Proposed brain rot places additional emphasis on passive/trivial content quality and a cognitive–emotional configuration; whether this adds explanatory value is unknown	Discriminant and incremental validity required
Internet addiction/problematic internet use	Broad problematic internet engagement across activities, often characterized by reduced control and impairment [16, 32]	Internet use across multiple activities	Reduced control, tolerance‐like patterns, and impairment	Broader than content quality and may involve gaming, communication, information seeking, or other activities; brain rot is hypothesized to focus on low‐quality passive content	Discriminant and incremental validity required
Technostress	Stress arising from technology‐related overload, complexity, uncertainty, invasion, or other technology demands [10, 38]	Technology demands, often occupational	Fatigue, cognitive strain, and frustration	Can arise from mandatory work systems such as EHRs without voluntary low‐quality recreational content consumption	Conceptually established; empirical overlap possible
Burnout	A work‐related syndrome associated with chronic occupational stress [27]	Chronic occupational stress	Exhaustion, cynicism, and reduced professional efficacy	Work‐specific and not defined by recreational digital‐content consumption; may coexist as a confounder or contextual condition	Conceptually established; empirical overlap possible

### 3.3. Step 5: Identifying a Model Case

Step 5 presents a model case, whereas Step 6 uses additional borderline, related, and contrary cases to clarify conceptual boundaries [26]. The cases are hypothetical conceptual illustrations rather than validated clinical or diagnostic presentations. Their purpose is to show how the proposed attributes may co‐occur and how the configuration differs from frequent controlled media use or technology‐related occupational strain.

#### 3.3.1. Model Case

“Maria is a 28‐year‐old emergency department nurse who works rotating 12‐h shifts. She initially began watching short‐form entertainment during breaks and after work to decompress. Over several months, her recreational use expanded into repeated scrolling before shifts and prolonged viewing after arriving home, sometimes delaying sleep. She describes mental fatigue and difficulty sustaining attention after long periods of scrolling, becomes irritable when trying to stop, and repeatedly reinstalls applications after attempts to reduce use. She recognizes that the pattern is displacing sleep, exercise, and offline activities, yet finds it difficult to disengage.”

Analysis: This model case illustrates the proposed five‐attribute configuration: recurrent passive low‐quality content consumption, cognitive disruption, emotional dysregulation, reduced control over engagement, and functional impairment. The apparent escalation provides a possible temporal course but is not itself a defining attribute. Because the case is hypothetical, it illustrates conceptual structure rather than demonstrating causality, prevalence, or diagnostic validity in nurses.

### 3.4. Step 6: Identifying Additional Cases

#### 3.4.1. Borderline Case

“Lena is a 31‐year‐old ICU nurse who watches short‐form cooking and lifestyle content for about 90 min on many evenings. She occasionally reports mental fatigue and irritability after prolonged viewing but can stop when needed; can forgo social media without distress; and reports no meaningful interference with sleep, relationships, or work. Her digital use has increased during a demanding staffing period, but she continues to choose when and how long she engages.”

Analysis: The borderline case includes passive consumption and possible cognitive/emotional disruption but lacks reduced control and functional impairment, showing why frequent use alone is insufficient.

#### 3.4.2. Related Case

“Khalid is a 40‐year‐old oncology nurse whose hospital recently implemented an EHR with extensive documentation requirements, frequent alerts, and continuous digital interaction. He reports fatigue, frustration, and difficulty concentrating during documentation, particularly late in the shift. Outside work, however, his digital use is limited and purposeful; he does not spend prolonged periods on short‐form recreational content and has no difficulty disengaging from social media.”

Analysis: This related case illustrates technostress arising from mandatory work‐related digital demands rather than the proposed voluntary low‐quality content pattern [10]. Fatigue and cognitive strain can overlap across the two concepts, but Khalid lacks the focal recreational exposure and reduced‐control pattern. The comparison also shows why EHR burden should be treated as a contextual work factor rather than a defining feature of brain rot.

#### 3.4.3. Contrary Case

“Sara is a 35‐year‐old emergency department nurse working in a technology‐intensive clinical environment. She uses recreational media deliberately and for limited periods, usually after completing planned activities. She can disengage without difficulty; does not allow scrolling to displace sleep, exercise, family interaction, or professional learning; and reports no persistent mental fatigue, irritability, or urge to continue using digital content when she intends to stop. Her attention, mood, relationships, and work functioning remain stable.”

Analysis: The contrary case lacks the proposed attributes and illustrates purposeful, controlled digital use without functional interference. It clarifies that digital exposure itself—even in a technology‐intensive workplace—is insufficient to constitute the proposed concept.

### 3.5. Step 7: Identifying Antecedents and Consequences

Antecedents are conditions preceding a concept [26]. Proposed antecedents include access to algorithmic feeds, habitual passive use, persuasive platform features, and recreational use for short‐term escape [16, 32, 35, 36]. Poor sleep or stress may affect self‐regulation but are not established causes. In nursing, EHR burden, long shifts, workload, emotional demands, and limited recovery are contextual vulnerabilities [7–10, 29].

To avoid circularity, cognitive disruption, emotional dysregulation, and functional impairment are not repeated as consequences because they are part of the proposed configuration. Potential downstream outcomes include displacement of restorative activities, poorer recovery, reduced professional learning and relational engagement, and impaired work functioning [16, 32, 37, 40]. Patient‐safety, burnout, turnover, and care‐quality effects remain untested nursing‐management hypotheses (Table 3).

**TABLE 3:** Antecedents and consequences of brain rot in nursing.

Level	Proposed antecedents/contextual vulnerabilities	Potential downstream outcomes to test
Behavioral/digital	Ready access to algorithmic feeds, habitual passive use, persuasive engagement features, and recreational use for short‐term escape or relief	Displacement of restorative offline activities, poorer recovery, and possible narrowing of sustained learning or leisure activities. Temporal ordering requires longitudinal testing
Individual/recovery	Stress, sleep/circadian disruption, and reduced recovery may influence self‐regulation or the appeal of low‐effort content; these are vulnerabilities rather than established causes	Reduced engagement in sleep, exercise, learning, or relationships may be examined as downstream outcomes rather than assumed effects
Nursing work context	EHR/alert burden, shift fatigue, workload, emotional demands, and limited recovery opportunities are contextual modifiers, not defining attributes or established causes	Reduced professional learning engagement, relational engagement, or work functioning should be tested after accounting for burnout, technostress, workload, and sleep
Organizational	Work environments that constrain recovery or intensify technology‐related strain may modify vulnerability but are not components of brain rot	Patient‐safety, care‐quality, burnout, turnover, liability, or workforce effects remain hypotheses and should not be inferred from the present concept analysis

### 3.6. Step 8: Defining Empirical Referents

Empirical referents are observable and measurable indicators used to determine whether the attributes of a concept are present [26]. For this provisional configuration, measurement should capture the five proposed attributes and, crucially, test whether they form a structure distinguishable from problematic social media use, internet addiction/problematic internet use, technostress, and burnout. Behavioral indicators may include frequency and passivity of low‐quality content consumption, difficulty disengaging, unsuccessful attempts to reduce use, and use that displaces recovery. Cognitive and emotional domains should be assessed with validated measures of sustained attention, subjective cognitive fatigue, and emotional regulation, while functional indicators should capture interference with sleep, learning, relationships, self‐care, or work.

No evidence‐based screen‐time threshold currently diagnoses brain rot, and no nursing‐specific instrument is validated for this purpose. Three brain‐rot scales have recently been reported [41–43], providing early attempts to operationalize the construct in non‐nursing populations. Their dimensional structure, psychometric performance, and applicability to nurses require independent evaluation. Existing measures of problematic smartphone or internet use and sleep [44, 45] may contribute to convergent and discriminant validation, but they should not be treated as diagnostic substitutes for brain rot. Nursing‐specific scale adaptation or development should follow confirmation of the construct’s dimensional structure rather than precede it.

## 4. Discussion

This analysis positions brain rot as a provisional and testable conceptual configuration rather than an established nursing disorder or occupational hazard. Its potential contribution is the hypothesis that recurrent passive consumption of low‐quality digital content may co‐occur with cognitive and emotional disruption, reduced control over engagement, and meaningful functional interference. The central validity question is whether this configuration adds explanatory value beyond problematic social media use and internet addiction, which already encompass reduced control and functional impairment [16, 32]. Until discriminant and incremental validity are demonstrated, brain rot should be considered an emerging conceptual label rather than a separate diagnosis.

### 4.1. Contributions Beyond Existing Brain Rot Literature

Brain‐rot scholarship is expanding rapidly, with emerging studies examining the construct predominantly among students, Generation Z, and other younger populations [19–22, 41–43, 46–48]. A 2026 scoping review of brain rot and low‐value social‐media exposure in adults [49] further synthesized emerging cognitive, emotional, and behavioral concerns associated with excessive consumption of low‐value digital content. This review was published at the close of the present study’s January–March 2026 search period and was not incorporated into the original 56‐source analytic corpus; however, its findings are considered here to situate the present analysis within the rapidly evolving literature.

The present study extends this emerging literature in a different direction. Rather than mapping available evidence, it applies Walker and Avant’s concept‐analysis framework to specify provisional defining attributes; clarify conceptual boundaries with problematic social media use, internet addiction, technostress, and burnout; and identify empirical referents. Its specific nursing‐management contribution is to distinguish voluntary recreational low‐quality content exposure from mandatory occupational technology and to propose testable relationships among personal media habits, recovery, EHR burden, shift fatigue, workload, and technostress. These proposed relationships remain theoretical and require nursing‐specific discriminant, incremental, and longitudinal validation.

Conceptual differentiation nevertheless remains challenging. Problematic social media use and internet addiction share reduced control and functional impairment with the proposed brain‐rot configuration, and existing measures may capture substantial overlapping variance. The proposed emphasis on passive, trivial, or low‐quality content together with cognitive–emotional disruption may offer additional explanatory value, but this requires factor‐analytic, discriminant‐validity, and incremental‐validity testing. If the proposed configuration does not demonstrate explanatory value beyond established problematic‐use constructs, a separate brain‐rot construct would have limited conceptual utility.

The proposed pathway also avoids assuming a specific neurobiological mechanism. Research on media multitasking and heavy internet use supports investigation of attention and executive control [23–25], while platform design and social feedback may help explain sustained engagement [35, 36]. These findings provide plausible mechanisms for investigation but do not demonstrate dopaminergic dysregulation or neurological deterioration caused by brain rot. Figure 3 therefore presents a provisional pathway in which contextual work vulnerabilities may interact with voluntary low‐quality digital exposure. Occupational digital load, shift fatigue, workload, and limited recovery may influence susceptibility or recovery but are not defining components or established causes of the concept. The five proposed defining attributes comprise passive low‐quality content consumption, cognitive disruption, emotional dysregulation, reduced control over engagement, and functional impairment. Possible escalation over time is treated separately as a course characteristic requiring longitudinal confirmation. Burnout and technostress may coexist but remain conceptually distinct.

**FIGURE 3:**
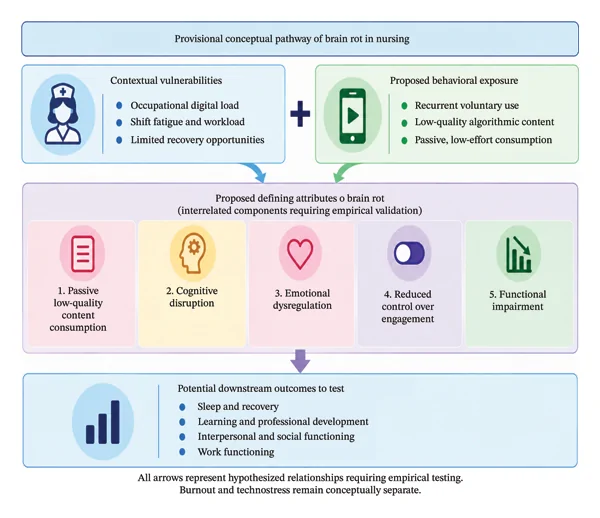
Provisional conceptual pathway of brain rot in nursing.

### 4.2. Implications for Nursing Management

Nurse managers can act on established digital‐work concerns without assuming that brain rot is present. Immediate priorities include reviewing modifiable EHR burden, unnecessary alerts, after‐hours connectivity, technology‐related strain, and opportunities for breaks, sleep, and recovery [9, 10]. Leaders can also support research that clearly separates voluntary recreational media use from mandatory professional technology exposure and tests, i.e., whether personal digital habits interact with workload, shift fatigue, sleep, and recovery.

Management support for education may emphasize digital competence, attention management, media literacy, intentional technology use, and critical appraisal of algorithmically curated content [24, 25, 50]. Brain rot may be discussed as an emerging concept, but it should not be presented to staff or students as a validated diagnosis or established occupational hazard. Practice and education should continue to rely on established approaches to cognitive recovery, healthy digital boundaries, and technology‐related well‐being while the construct is evaluated.

Brain rot–specific screening, surveillance, manager training, organizational policy, or intervention is premature. Such actions should await evidence of dimensional structure, reliable measurement, discriminant and incremental validity against problematic social media use, internet addiction, technostress, burnout, and related constructs and meaningful prediction of nursing outcomes. Policy and workforce initiatives should therefore address established concerns such as EHR usability, alert burden, recovery time, and responsible after‐hours connectivity while supporting rigorous validation research.

### 4.3. Strengths and Limitations of the Study

Strengths include structured use of Walker and Avant’s method and explicit boundary analysis. Limitations include scarce nursing‐specific evidence, reliance on non‐nursing populations, purposive searching without formal risk‐of‐bias scoring, unresolved overlap with problematic digital use, and unproven causal ordering, temporal progression, and nursing‐specific measurement.

Future research should test dimensional structure, reliability, discriminant and incremental validity, and longitudinal ordering in nursing populations, comparing brain‐rot measures with problematic social media use, internet addiction, technostress, burnout, sleep, and cognitive fatigue [9, 10, 27, 37]. Multisite studies should separate personal digital exposure from mandatory work technology and account for work environment [38, 51].

Validation should avoid assuming that all digital exposure is harmful. Nursing self‐care literature supports recovery [52]; concerns about shallow online engagement have been raised [53], while longitudinal evidence in older adults reports potential benefits of regular internet use [54]. The label has also been applied metaphorically to artificial‐intelligence systems [55], reinforcing the need for a human‐specific definition. Shift‐related safety evidence [56] highlights fatigue as a confounder, and general cognitive screens such as the Montreal Cognitive Assessment [57] are not brain‐rot diagnostic tests.

## 5. Conclusion

Brain rot remains a provisional and evolving concept rather than an established nursing diagnosis or occupational hazard. This concept analysis proposes a five‐attribute configuration comprising passive low‐quality content consumption, cognitive disruption, emotional dysregulation, reduced control over engagement, and functional impairment. Although these attributes provide a preliminary framework for conceptualizing brain rot, their distinctiveness from established constructs—particularly problematic social media use, problematic internet use, technostress, and burnout—requires empirical examination. Future research should therefore establish construct, discriminant, and incremental validity and determine whether the proposed configuration provides explanatory value beyond existing measures of problematic digital‐media use.

For nursing management, the concept should currently be viewed as a research and workforce–well‐being consideration rather than a basis for screening, labeling, or intervention. Nurse leaders can continue to address established digital‐work challenges, including technostress, EHR burden, excessive connectivity, sleep disruption, workload, and inadequate recovery opportunities, while supporting research into how recreational digital‐media behaviors may interact with these occupational conditions. Brain rot–specific assessment tools, organizational policies, or interventions should be considered only after the construct has demonstrated reliable measurement, empirical validity, and meaningful relevance to nursing outcomes.

**Nomenclature**
ADHD: Attention‐deficit/hyperactivity disorder
EHR: Electronic health record

## Author Contributions

Ebtsam Aly Abou Hashish: conceptualization, methodology, supervision, formal analysis, investigation, writing–original draft, and writing–review and editing.

Reham Yasin: conceptualization, methodology, formal analysis, investigation, writing–original draft, writing–review and editing.

## Funding

This research did not receive any specific grant from funding agencies in the public, commercial, or not‐for‐profit sectors.

## Ethics Statement

This concept analysis synthesized published and publicly available literature and involved no participant recruitment, identifiable private information, or primary data collection. Ethical approval was obtained from King Abdullah International Medical Research Center, Jeddah, Saudi Arabia (NRJ25/060/12).

## Consent

Consent was not applicable because the study did not involve human participants or identifiable patient information.

## Conflicts of Interest

The authors declare no conflicts of interest.

## Data Availability

No primary dataset was generated or analyzed in this concept analysis. The literature and sources supporting the analysis are identified in the reference list. Additional details of the literature‐charting process are available from the corresponding author on reasonable request.
